# Comparative Analysis of Vaginal and Abdominal Uterine Manipulation in Laparoscopic Hysterectomy: The Boztosun Technique

**DOI:** 10.3390/jcm14113652

**Published:** 2025-05-23

**Authors:** İsmail Çelik, Abdullah Boztosun, Fatma Ceren Güner

**Affiliations:** Department of Gynecology Obstetrics, Akdeniz University, Antalya 07070, Turkey; ismailcelikk1994@gmail.com (İ.Ç.); abdullahboztosunyrd@hotmail.com (A.B.)

**Keywords:** Boztosun technique, intra-abdominal manipulation, laparoscopic hysterectomy, minimally invasive gynecology, Rein technique, uterine manipulator

## Abstract

**Background/Objectives**: Laparoscopic hysterectomy is commonly performed for benign gynecologic conditions, and the choice of uterine manipulation technique may influence surgical outcomes. The Boztosun technique, a modification of the classical Rein technique, enables intra-abdominal manipulation of the uterus without requiring transvaginal instruments. This study aimed to compare the Boztosun technique with a conventional vaginal uterine manipulator in terms of surgical efficiency and perioperative outcomes. **Methods**: This retrospective study included 30 patients who underwent laparoscopic hysterectomy for benign indications at Akdeniz University Hospital between March 2022 and March 2024. Fifteen patients underwent surgery using the Boztosun technique, and fifteen with a vaginal uterine manipulator. Operative time, colpotomy time, manipulator placement time, hospital stay, uterine weight, hemoglobin change, and complications were compared. **Results**: The Boztosun technique was associated with significantly shorter operative time (81.67 ± 11.02 min vs. 109 ± 10.85 min, *p* < 0.001), colpotomy time (4.13 ± 0.92 min vs. 8.87 ± 0.92 min, *p* < 0.001), manipulator placement time (0.81 ± 0.27 min vs. 8.07 ± 1.22 min, *p* < 0.001), and hospital stay (2.13 ± 0.35 days vs. 3.53 ± 0.92 days, *p* < 0.001). No significant differences were found in uterine weight, hemoglobin decrease, or complication rates. All procedures were completed laparoscopically without conversion to open surgery. **Conclusions**: The Boztosun technique may serve as a safe, efficient, and cost-effective alternative to vaginal uterine manipulators in laparoscopic hysterectomy. Its simplified intra-abdominal approach offers advantages in surgical workflow and recovery. Further prospective studies are needed to validate these findings and assess their applicability in broader clinical settings.

## 1. Introduction

Hysterectomy remains one of the most common major gynecologic surgeries worldwide and is a key treatment option for many benign and malignant conditions. In the United States, approximately 400,000 to 500,000 hysterectomies are performed annually, the majority of which address benign pathologies such as fibroids, abnormal uterine bleeding, and endometriosis [1]. Comparable findings have been reported in other countries, where large population-based studies and national health databases have documented hundreds of thousands of hysterectomies over the past decade [2,3]. These consistent trends highlight the widespread and enduring clinical relevance of hysterectomy and reinforce the need to continually refine surgical techniques, particularly within the framework of minimally invasive gynecologic surgery. In parallel, a growing global trend toward minimally invasive approaches has been observed, with population-level data showing that more than half of hysterectomies are now performed using laparoscopic or robot-assisted techniques in several healthcare systems [4].

This procedure can be performed through open surgery or minimally invasive techniques. In laparoscopic hysterectomy, uterine manipulators are frequently used to improve exposure by distancing the uterus from surrounding structures such as the ureters [5]. These devices usually include a cervical cup that helps define the colpotomy line and maintain pneumoperitoneum during surgery [6]. While they are designed to improve safety and assist with anatomical orientation, both disposable and reusable manipulators can be expensive, technically demanding, and occasionally limited in adaptability [5,6].

In 2018, Boztosun et al. introduced the Rein technique, an intra-abdominal method of uterine manipulation based on the idea of guiding the uterus like a bridle on a horse [7]. This approach removed the need for vaginal instruments but had some practical challenges, particularly with how the cotton tape was applied and how stable it remained during surgery [7]. To address these issues, the technique was gradually refined over time. This modified version eventually evolved into what we now refer to as the Boztosun technique. It allows for better control of the uterus, requires less assistance, and eliminates the need for transvaginal components.

The purpose of this study was to compare the Boztosun technique with traditional vaginal uterine manipulators in terms of surgical performance, efficiency, and patient recovery in laparoscopic hysterectomy.

## 2. Materials and Methods

This retrospective study was approved by the Clinical Research Ethics Committee of Akdeniz University (Approval No: TBAEK-188) and conducted in accordance with the principles of the Declaration of Helsinki. All procedures were carried out at the Department of Obstetrics and Gynecology, Akdeniz University Hospital.

**Figure 1:**
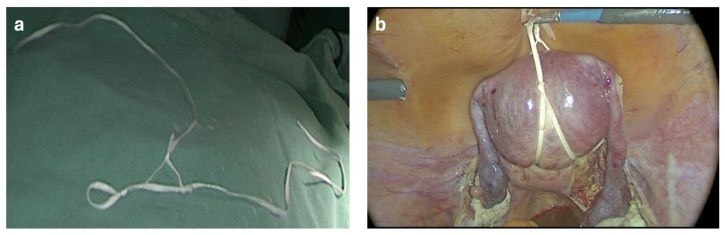
Classical Rein technique for uterine manipulation. (**a**) Cotton tape prepared on the surgical field prior to abdominal insertion. (**b**) Intra-abdominal appearance of the uterus manipulated using the classical Rein technique [7].

**Figure 2:**
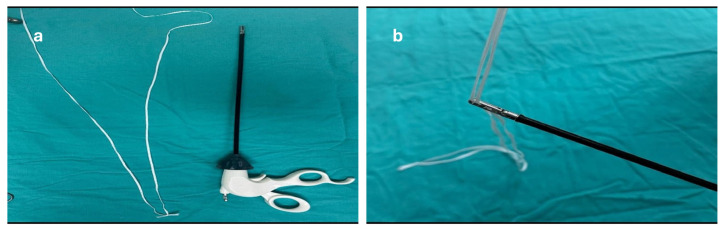
Preparation steps of the Boztosun technique. (**a**) Cotton tape arranged and laid out alongside a standard uterine manipulator before the procedure. (**b**) Looping of the cotton tape through the jaws of the grasper for intra-abdominal insertion.

Patients were not randomized. All surgeries were performed by the same experienced gynecologic surgical team. The Boztosun technique, a simplified and more practical version of the previously described Rein technique, was gradually developed and incorporated into routine clinical practice [7]. The classical Rein technique, which served as the basis for the Boztosun modification, is shown in Figure 1. The preparation steps of the Boztosun technique, including the looping of cotton tape through the jaws of the grasper, are illustrated in Figure 2.

For this study, patients who had undergone laparoscopic hysterectomy using either the Boztosun technique or a conventional vaginal uterine manipulator were identified through a review of electronic medical and surgical records. The study included 30 patients operated on for benign indications between March 2022 and March 2024. Of these, 15 underwent surgery with the Boztosun technique and 15 with a conventional vaginal manipulator (V-Care®, CONMED Corporation, Utica, NY, USA).

**Figure 3:**
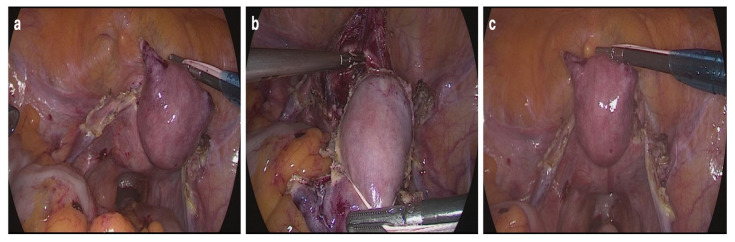
Uterine manipulation achieved using the Boztosun technique (modified Rein technique). (**a**) Lateral traction of the uterus during dissection. (**b**) Anterior traction and bladder flap dissection. (**c**) Posterior traction and visualization of posterior uterine structures.

In the Boztosun group, two cotton tapes were passed through the jaws of a grasper and inserted into the abdomen through a 5 mm trocar. A second grasper was used to tighten and position the loop around the uterus. The tape was then fixed externally, allowing uterine manipulation using a single grasper, which served as the only instrument for this purpose throughout the surgery. This approach helped avoid several challenges associated with the classical Rein technique, such as knot tying inside the abdomen, slippage of the tape, or its adherence to surrounding tissue due to blood exposure [7]. Intraoperative images showing lateral, anterior, and posterior traction of the uterus using the Boztosun technique are presented in Figure 3 and illustrate how this approach ensures clear visualization during dissection.

The collected data included demographic variables (age, BMI, menopausal status, and history of abdominal surgery), surgical parameters (colpotomy time, operative time, manipulator placement time, uterine weight, and change in hemoglobin), and recovery outcomes (length of hospital stay, complications, and conversion to open surgery).

Statistical analysis was performed using IBM SPSS Statistics for Windows, Version 23.0 (IBM Corp., Armonk, NY, USA). The normality of the data was assessed with the Shapiro–Wilk test, Q–Q plots, and evaluation of skewness and kurtosis. Depending on data distribution, continuous variables were compared using either the independent *t*-test or the Mann–Whitney U test. Categorical variables were analyzed using Fisher’s exact test. A *p*-value below 0.05 was considered statistically significant. A power analysis based on the observed difference in operative time (Cohen’s d = 2.51) showed that a sample size of four patients per group would be sufficient to achieve 80% power at a significance level of 5%. Therefore, the total sample of 30 patients (15 per group) was considered statistically adequate for the primary outcome.

## 3. Results

A total of 30 patients were included in the study, with 15 in the Boztosun group and 15 in the vaginal manipulator group. The mean age was 49.4 ± 5.05 years in the Boztosun group and 47.6 ± 8.24 years in the vaginal group (*p* = 0.145). The two groups were also similar in terms of body mass index (BMI) (*p* = 0.848), menopausal status (*p* = 0.705), and prior abdominal surgery (*p* = 0.713). A detailed comparison of perioperative characteristics is provided in Table 1.

The Boztosun technique was associated with significantly shorter operative time (81.67 ± 11.02 min vs. 109 ± 10.85 min, *p* < 0.001), hospital stay (2.13 ± 0.35 days vs. 3.53 ± 0.92 days, *p* < 0.001), colpotomy time (4.13 ± 0.92 min vs. 8.87 ± 0.92 min, *p* < 0.001), and manipulator placement time (0.81 ± 0.27 min vs. 8.07 ± 1.22 min, *p* < 0.001). These findings, summarized in Table 1, suggest a clear operative advantage of the Boztosun technique in terms of surgical efficiency and recovery time.

No statistically significant differences were observed between groups regarding uterine weight (207.87 ± 99.09 g vs. 223.4 ± 126.08 g, *p* = 0.710) or hemoglobin decrease (2.00 ± 0.65 g/dL vs. 2.21 ± 0.56 g/dL, *p* = 0.309), indicating a comparable surgical burden in both approaches.

All procedures were completed laparoscopically without conversion to open surgery. No intraoperative or postoperative complications were reported, and no adverse events occurred during the 30-day postoperative follow-up period.

**Figure A1:**
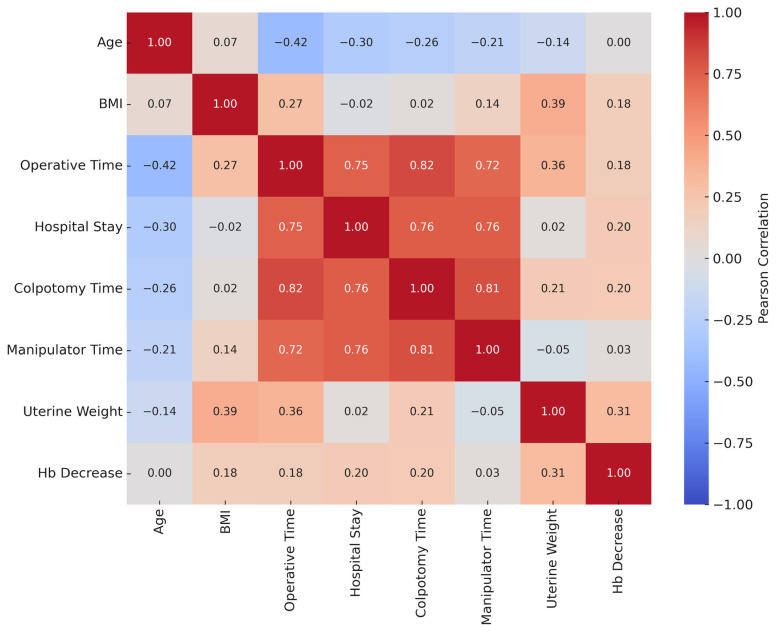
Correlation heatmap of continuous variables.

Operative time was strongly correlated with hospital stay, colpotomy duration, and manipulator placement time. Additionally, a negative correlation was observed between patient age and operative time (r = −0.415, *p* = 0.023), suggesting that younger patients tended to have longer surgeries. These relationships are presented in Table 2 and visualized in Appendix A Figure A1 as a correlation heatmap.

## 4. Discussion

This study demonstrates that the Boztosun technique, an intra-abdominal approach to uterine manipulation, offers clear advantages over traditional vaginal manipulators in laparoscopic hysterectomy. Patients in the Boztosun group experienced significantly shorter operative times, faster instrument application, reduced colpotomy duration, and earlier hospital discharge. These findings align with the intended purpose of the technique, which is to improve surgical efficiency while minimizing the reliance on complex or costly vaginal devices [8].

In their comparative analysis, Mettler and Nikam reported that certain single-use uterine manipulators may be associated with higher cost and potential issues in assembly or intraoperative stability during laparoscopy [8]. This supports the practical benefits observed with the Boztosun technique, particularly in terms of simplicity and cost-effectiveness.

Furthermore, our results are consistent with earlier studies emphasizing the critical role of uterine manipulation in optimizing surgical exposure during laparoscopic hysterectomy. For example, Puntambekar et al. demonstrated that hitching the uterus to the abdominal wall improves stability and allows safer dissection by enhancing visualization, while van den Haak et al. and Abdel Khalek et al. noted that poor visualization or inappropriate use of manipulators may prolong operative time and reduce procedural safety in complex cases [9,10,11].

The Boztosun method eliminates the need for a transvaginal device, thereby avoiding potential complications such as vaginal laceration, loss of pneumoperitoneum, or disruption of cervical integrity. Previous studies have also reported that the use of vaginal manipulators may be limited in patients with altered pelvic anatomy, including those with prior pelvic surgery or vaginal stenosis [10,11]. Moreover, high BMI, previous cesarean section, and the presence of endometrioma have been identified as significant preoperative predictors of increased procedural difficulty in laparoscopic hysterectomy, further supporting the relevance of an intra-abdominal approach in such cases [12].

Our findings support the idea that intra-abdominal cotton tape manipulation can offer more dynamic and flexible control. By enabling controlled lateral, anterior, and posterior uterine traction (as illustrated in Figure 3), the Boztosun technique may facilitate adequate exposure of the vaginal cuff during colpotomy, thereby offering a functional alternative to the delineation typically provided by a vaginal cup. Similar concepts were explored by Puntambekar et al., who described an internal manipulator designed to reduce dependence on vaginal components [9]. Moreover, van den Haak et al. noted that vaginal devices may be challenging or contraindicated in cases of cervical stenosis, vaginal atrophy, or abnormal anatomy [10]. In such situations, the Boztosun technique may offer a practical and safer alternative.

In oncologic gynecologic surgery, the safety of intrauterine manipulators remains controversial. Several studies have raised concerns about their potential role in tumor dissemination. In a large multicenter cohort, Padilla-Iserte et al. reported higher recurrence rates and decreased disease-specific survival among patients with early-stage endometrial cancer undergoing laparoscopic hysterectomy with intrauterine manipulators compared to those without [13]. Zorzato et al., in a recent meta-analysis involving over 5000 patients, did not find a statistically significant association between intrauterine manipulator use and recurrence or survival, though the hazard ratio for recurrence approached significance (HR: 1.52, 95% CI: 0.99–2.33, *p* = 0.05) [14]. Moreover, a prospective study by Sallée et al. revealed that intrauterine manipulator-related uterine perforation occurred in 11% of cases and was associated with a significantly higher rate of lymphovascular space invasion (67% vs. 25%, *p* = 0.02), which in turn influenced adjuvant treatment decisions in nearly one-quarter of cases [15]. Although our study focused exclusively on benign indications, these data underscore the importance of exploring manipulator-free techniques. The Boztosun technique, by avoiding transvaginal and intrauterine instrumentation, may offer theoretical oncologic advantages, especially in borderline or high-risk cases.

In our study, the Boztosun technique was associated with shorter operative time, fewer complications, and faster recovery. Although hemoglobin change and uterine weight were comparable, the elimination of transvaginal instrumentation and simpler manipulation setup may have contributed to faster recovery and shorter hospitalization. These benefits likely stem from the improved visualization and simplified setup made possible by direct intra-abdominal control. While the earlier Rein technique demonstrated feasibility, the Boztosun modification enhances stability, eliminates the need for intra-abdominal knot tying, and reduces the requirement for additional assistance, as reflected in the shorter manipulator placement time [7].

Correlation analysis in our cohort confirmed the clinical relevance of surgical efficiency. Operative time was positively correlated with hospital stay, colpotomy duration, and manipulator setup time. Interestingly, younger patients in our study had longer operative durations (r = −0.415, *p* = 0.023), although the underlying factors for this association remain uncertain and warrant further investigation.

Cost is another important consideration. The Boztosun technique relies on inexpensive materials such as standard cotton tape and a reusable grasper. In contrast, commercial vaginal manipulators are generally single-use and substantially more expensive due to their complexity. Mettler and Nikam highlighted similar concerns, noting that many commercially available single-use uterine manipulators tend to be expensive and may pose difficulties in terms of assembly and intraoperative stability [8]. The cost-effectiveness of the Boztosun approach may make it especially useful in resource-limited settings.

Finally, van den Haak et al. reviewed the performance of several uterine manipulators and noted that, despite their widespread use, the available evidence regarding their effect on visualization and surgical outcomes remains inconclusive [10]. Some designs may lack sufficient articulation or clear delineation of anatomical landmarks. In this context, the Boztosun technique, by providing direct intra-abdominal control, may help address some of these limitations and allow for more adaptable manipulation in select surgical scenarios.

Despite its promising results, this study has several limitations. The technique was developed and applied by a single experienced surgical team, and its generalizability to less experienced settings is unknown. Patient-reported outcomes, such as postoperative pain, cosmetic satisfaction, and recovery of daily activity, were not evaluated. Long-term effects on vaginal cuff healing and pelvic support were also beyond the scope of this study. In addition, the small sample size limits subgroup analysis and the study design lacked randomization, introducing a risk of selection bias. However, a post hoc power analysis confirmed that the study was adequately powered to detect differences in operative time, which was the primary endpoint.

## 5. Conclusions

The Boztosun technique appears to be a safe, effective, and cost-efficient alternative to conventional vaginal uterine manipulators in laparoscopic hysterectomy. In this retrospective study, the method significantly reduced operative time, hospital stay, and manipulator placement time, without increasing intraoperative risk. Its intra-abdominal approach offers several practical advantages, particularly in cases where vaginal access is limited or not preferred. These findings support further evaluation of the technique in larger, prospective, randomized studies to confirm its clinical and logistical benefits. Future research should also assess its impact on patient-reported outcomes and its applicability across different levels of surgical experience.

## Figures and Tables

**Table 1:** Comparison of perioperative outcomes between groups.

Variable	Boztosun Group (*n* = 15)	Vaginal Manipulator (*n* = 15)	*p*-Value
Age (years)	49.4 ± 5.05	47.6 ± 8.24	0.145
BMI (kg/m^2^)	29.0 ± 4.8	28.65 ± 5.19	0.848
Operative time (min)	81.67 ± 11.02	109.0 ± 10.85	<0.001
Hospital stay (days)	2.13 ± 0.35	3.53 ± 0.92	<0.001
Colpotomy time (min)	4.13 ± 0.92	8.87 ± 0.92	<0.001
Manipulator placement time (min)	0.81 ± 0.27	8.07 ± 1.22	<0.001
Uterine weight (g)	207.87 ± 99.09	223.4 ± 126.08	0.710
Hemoglobin decrease (g/dL)	2.00 ± 0.65	2.21 ± 0.56	0.309
Menopausal status, *n* (%)	5 (33.3%)	4 (26.7%)	0.705
Prior abdominal surgery, *n* (%)	6 (40%)	5 (33.3%)	0.713

**Table 2:** Significant correlations among continuous perioperative variables.

Variable 1	Variable 2	Pearson Correlation Coefficient (r)	*p*-Value
Operative time	Age	−0.415	0.023
Operative time	Hospital stay	0.745	<0.001
Operative time	Colpotomy time	0.824	<0.001
Operative time	Manipulator placement time	0.723	<0.001

## Data Availability

The datasets used or analyzed during the current study are available from the corresponding author, F.C.G., on reasonable request.

## Abbreviations

The following abbreviations are used in this manuscript:

BMI	Body mass index
G	Grams
g/dL	Grams per deciliter
kg/m^2^	Kilograms per square meter
min	Minutes
mm	Millimeters
SD	Standard deviation

## Appendix A

Correlation heatmap of continuous perioperative variables. Pearson correlation coefficients are presented for all pairwise comparisons. Strong positive and negative associations are color coded from blue to red.
